# A dream or reality: Consideration of ‘bloodless’ hematopoietic stem cell transplants for Jehovah’s witness patients

**DOI:** 10.1016/j.htct.2025.103958

**Published:** 2025-07-30

**Authors:** Michael MacNeill, Adrienne Fulford, Deanna Caldwell, Uday Deotare

**Affiliations:** a Department of Medicine, Schulich School of Medicine and Dentistry, Western University, London, ON, Canada; b London Regional Cancer Program, London Health Sciences Centre, London, ON, Canada; c Victoria Hospital, London Health Sciences Centre, London, ON, Canada; d Division of Hematology, Department of Medicine, Schulich School of Medicine and Dentistry, Western University, London, ON, Canada; e The Centre for Quality, Innovation and Safety, Department of Medicine, Schulich School of Medicine and Dentistry, Western University, London, ON, Canada

**Keywords:** Jehovah’s witness, Autologous hematopoietic stem cell transplantation, Non-hodgkin’s lymphoma, Multiple myeloma, Transfusion

## Abstract

**Background:**

Hematopoietic stem cell transplantation (HSCT) is an important part of treatment for many hematologic conditions. The high-dose chemotherapy used in HSCTs puts patients at risk of significant cytopenias which often necessitate blood product transfusions. Certain populations, including Jehovah’s Witnesses, are unable to receive blood product transfusions during their transplant and thus, in the past, they have been seen as unsuitable candidates for transplantations. However, there has been growing evidence of the safety and efficacy of so-called “bloodless” HSCT protocols.

**Methods:**

The most recent and relevant literature on “bloodless” transplants were identified through Embase, MEDLINE, and PubMed, and analyzed to construct a “bloodless” HSCT protocol at a Canadian centre. Since 2021, the regimen was utilized for four autologous transplantations in three different Jehovah’s Witness patients.

**Results:**

None of the patients had a significant bleeding event nor a hemoglobin nadir below 8.0 g/dL. Minor bleeding events, predominantly mucositis, resolved with site-specific management. No patient had significant thrombocytopenia, and all the cell lines of patients had normalized without transfusions by the time of discharge. All patients were hospitalized for <30 days, similar to the experience of the centre with “regular” autologous transplants.

**Conclusion:**

Careful planning and tailored regimens support the achievability of “bloodless” HSCTs in patients, such as Jehovah’s Witnesses, allowing practitioners to provide care to a previously excluded group and minimize the use of blood products in all HSCT patients.

## Introduction

Hematopoietic stem cell transplantation (HSCT), both autologous and allogeneic, is a key pillar in the treatment of many non-malignant and malignant hematologic conditions.^1^ Protocols for HSCT rely on high-dose chemotherapy which often leads to the complication of severe pancytopenia. In these instances, typical hematopoietic support for HSCT patients utilizes transfusion of blood products to manage possible life-threatening cytopenias. For example, most institutions initiate red blood cell transfusions at hemoglobin concentrations of <7.0 g/dL and platelet transfusions for platelet counts <10 × 10^9^ cells/L in non-bleeding patients.^2^ These are the values at which patients are at increased risk of end organ dysfunction and spontaneous intracranial hemorrhage, respectively.^3^ The transfusion requirements for HSCTs can often be quite high. For example, on average, it has been reported that allogeneic HSCT recipients necessitate a mean of 6.8 units of packed red blood cells within the first 60 days alone.^4^ Given this frequent necessity of transfusions in HSCTs, patients who refuse blood product transfusions have previously not been candidates for HSCT. This has left certain groups, particularly the approximately 8.5 million Jehovah’s Witnesses worldwide,^5^ marginalized, and unable to access standard-of-care treatment for conditions with high morbidity and mortality.^6^

While there has been an increase in recent years in attempts to minimize transfusion products and optimize “bloodless” HSCTs,^7^ there remains a scarcity of descriptions of the implementation of “bloodless” protocols, particularly in Canada. This study sought to assess the feasibility and safety of a “bloodless“ HSCT protocol at a Canadian centre for Jehovah Witness patients based on the most relevant literature.

## Methods

Relevant literature describing “bloodless” HSCT protocols were identified through Embase, MEDLINE and PubMed databases. The first reported “bloodless” HSCT was in 1996.^8^ In total, ten relevant articles were identified which described “bloodless” protocols. While there remains no consensus guidelines for “bloodless” transplantations, general principles were identified. These specialized protocols have also been shown to be quite effective with good overall patient outcomes.^7^, ^8^, ^9^, ^10^, ^11^, ^12^, ^13^, ^14^, ^15^, ^16^ Assessing these recent and relevant articles, London Health Sciences Centre’s (LHSC) Hematology Division was able to construct a “bloodless“ HSCT protocol based on their core principles and features (Table 1).

**Table 1:** London health sciences centre ‘bloodless’ hematopoietic stem cell transplant regimen.

Prerequisites & informed consent	
• Ideal parameters: Hb >11.0 g/dL, Plt >150 × 10^9^ cells/L	
• Acceptable parameters: Hb >9.0 g/dL, Plt >100 × 10^9^ cells/L	
• 10 % risk of overall mortality from bleeding/anemia/infections	
• CNS involvement increases risk of fatal intracranial hemorrhage (10 % risk)	
• Informed documented consent of refusal of blood product	
Pre-transplant optimization	
• Eprex 40,000 units SC q1wk. Target Hb ≥11.0 g/dL. Discontinue if Hb ≥12.0 g/dL	
• Iron sucrose 300 mg IV over two hours q1wk. Target Hb ≥10.0 g/dL. Discontinue if Hb ≥12.0 g/dL	
• If female patient, consider: oral contraceptive, or Medroxyprogesterone IM injection	
During transplant hematopoietic support	
Medication support	• No aspirin/NSAIDS
	• Vitamin K 10 mg PO q.d. Monday, Wednesday, and Friday
	• Folic acid 5 mg PO q.d.
	• Vitamin B12 1,000 mg PO q.d.
	• Polyethylene glycol 3350 17 g PO q.d. (can increase if constipation develops)
	• Lansoprazole 30 mg PO b.i.d.
	• Saline nasal spray 1 spray each nostril t.i.d.
	• Filgrastim 5 mg/kg (300/480 mg) SC q.d., start Day +5
	• Eltrombopag 50–150 mg q.d. or romiplostim 4 mg/kg SC q1wk starting Day +5 until Plt >100 × 10^9^ cells/L
	• fluconazole (Diflucan) 400 mg PO or IV q.d. start Day 0
	• Acyclovir 400 mg PO or 250 mg IV every 12 h, start Day 0
	• Levofloxacin 500 mg PO q.d., start Day 0; when febrile, stop levofloxacin and start imipenem 500 mg IV q6h
	• Acetaminophen 650 mg PO q4h if febrile
	• IV fluids to ensure euvolemic status
	• If female patient: continue oral contraceptive if started pretransplant
Blood conservation	• Only twice weekly blood (Monday and Thursday)
	• CBC, LFTs, creatinine, electrolytes (+ PT, INR, fibrinogen once weekly)
	• Use pediatric vacutainers-MICROTAINERS for sampling (total volume = 0.25+ 0.4 + 0.4 + 0.4 = 1.5 mL each draw [1.8 mL for coagulation studies once weekly])
	• Do not use central line for blood draw
	• If fever, blood culture x2 – one central venous catheter, peripheral venipuncture only once/week (Use Bactec Adult/F bottles-10 mL x2)
	• If clinical unstable or other collections indicated, then above collection rule exempted
Anemia	• If Hb <11.0 g/dL, iron sucrose 300 mg IV q1wk; discontinue when Hb >12.0 g/dL
	• If Hb <10.0 g/dL, Eprex® 40,000 units SC q1wk; discontinue when Hb >12.0 g/dL
	• If Hb < 8.0 g/dL, then supplemental O_2_ 2 L/min by nasal prongs
Thrombocytopenia	• If Plt <30 × 10^9^ cells/L, tranexamic acid 1 g PO q4h
	• If Plt <10 × 10^9^ cells/L, tranexamic acid 4 g IV q4h
Active bleed	• Local hemostasis/pressure dressing
	• Change PO lansoprazole to pantoprazole 40 mg IV q12h if GI bleed
	• Tranexamic acid 1 g mouth rinse swish and spit QID for mucosal bleed
	• If systemic bleed, tranexamic acid 1 g IV q4h (avoid if hematuria)
	• If minor bleed, desmopressin IV 0.3 mg/kg IV q12H for 3 days
	• If continued bleeding after desmopressin, can consider Factor VIIa 90 mg/kg IV q2h until bleeding subsides
	• For epistaxis, xylometazoline (Otrivin®)/saline nasal spray two sprays in each nostril q15 min until bleed resolves

Hb: hemoglobin; Plt: platelets; PO: oral; SC: subcutaneous; IV: intravenous; q.d.: once daily; b.i.d.: twice daily; t.i.d.: three times daily; q: every; wk: week; h: hour; min: minute; CNS: Central nervous system; NSAIDS: non-steroidal anti-inflammatory drugs; CBC: complete blood count; LFTs: liver function tests; PT: prothrombin time; INR: international normalized ratio.

Management strategies for HSCT-related cytopenias were divided into two general categories: pre-transplant optimization and post-transplant management. Pre-transplant optimization involves targeting safe cell count levels prior to the conditioning therapy with these levels being achieved by erythropoietin agonists, intravenous (IV) iron and limiting blood loss.^8^, ^9^, ^10^ We utilized pre-transplant cell count targets for hemoglobin of >11.0 g/dL and for platelets >150 × 10^9^ cells/L (Table 1) with similar thresholds generally used in other studies.^8^, ^9^, ^10^ These thresholds were primarily based on retrospective assessments of pre-transplant values which led to more severe outcomes,^9^ while still maintaining the principles of a “transfusion-restrictive” approach and limiting any sequelae of increased viscosity at higher hematocrits.^2^ To achieve these cell counts, patients received regular erythropoietin stimulating agent (ESAs) injections, a strategy also commonly used for Jehovah’s Witnesses perioperatively or when critically ill.^17^ Specifically, our protocol used 40,000 units subcutaneously (SC) weekly of Eprex® (Epoetin alfa). In conjunction, erythropoiesis was further supported with IV iron supplementation,^8^, ^9^, ^10^ with our regimen using IV iron sucrose 300 mg every week. Additionally, to further prevent unnecessary menstrual blood loss, hormonal contraceptives have been used in various protocols,^8^, ^9^, ^10^ therefore, for women of childbearing age, we provided the option between an oral hormonal contraceptive or an intramuscular medroxyprogesterone injection to control menstruation. ESAs and intravenous iron were discontinued when hemoglobin reached >12.0 g/dL (Table 1).

Management is more complex post-transplant with prophylactic hematopoietic support, bleeding prevention, minimizing unnecessary blood loss, as well as reactive management for severe cytopenias and significant bleeding (Table 1). Prophylactic nutrient supplements have been utilized in a variety of “bloodless” regimens to support hematopoiesis.^8^, ^9^, ^10^^,^^12^^,^^13^ At LHSC, patients were given folic acid 5 mg oral (PO) once daily (q.d.) and vitamin B12 1,000 mg PO q.d., in addition to vitamin K 10 mg PO q.d. on Mondays, Wednesdays, and Fridays to prevent coagulopathies. Further hematopoietic support was given to improve platelet and neutrophil counts as these cell lines are often profoundly depleted during HSCT conditioning. It has become common to provide scheduled thrombopoietin receptor agonists (TPO-RAs) and granulocyte colony-stimulating factor (G-CSF) post-transplant when patients are expected to reach their cell count nadir.^8^, ^9^, ^10^^,^^12^^,^^13^ We administered filgrastim 300 mg, or 480 mg if patient weight was greater than 90 kg, SC q.d. starting on Day +5 after the transplant until the absolute neutrophil count reached 1.0 × 10^9^ cells/L and romiplostim 4 mg/kg SC weekly starting on Day +5 until the platelet count reached >100 × 10^9^ cells/L.

To prevent unnecessary blood loss, other regimens sought to prevent upper and lower gastrointestinal bleeding, and epistaxis,^8^, ^9^, ^10^^,^^12^^,^^13^ so our patients were given lansoprazole 30 mg PO twice daily (b.i.d.), PEG3350 17 g PO q.d., and saline nasal spray one spray per nostril q.d. A major source of blood loss in HSCT patients is the regular, at least daily, blood draws. Therefore, based on similar protocols in other studies,^8^, ^9^, ^10^, ^11^, ^12^, ^13^, ^14^, ^15^, ^16^ bloodwork was reduced from daily to twice per week and pediatric-sized vacutainers were used. Additionally, in the event of a fever, given post-transplant infections are another common risk of HSCTs, blood cultures were limited to only once per week. These blood conservation techniques have been estimated to reduce the average blood drawn from 40 mL per day to approximately 3 mL.^16^

If severe anemia or thrombocytopenia were to arise, various protocols increased hematopoietic support in addition to antifibrinolytic agents, achieving significant success.^7^, ^8^, ^9^, ^10^, ^11^, ^12^ For our protocol, when the patient’s hemoglobin was <11.0 g/dL, we initiated IV iron sucrose (300 mg weekly) and when their hemoglobin was <10.0 g/dL they were started on Eprex® (40,000 units SC weekly). If their platelets were less than 30 × 10^9^ cells/L, they were started on tranexamic acid (TXA - 1 g PO every four hours), which was increased to 4 g IV every four hours if their platelets dropped below 10 × 10^9^ cells/L.

Finally, in the event of bleeding, previous protocols implemented various systemic and local therapies to gain hemostasis.^7^, ^8^, ^9^, ^10^, ^11^, ^12^, ^13^, ^14^, ^15^, ^16^ For minor hemorrhages, defined as localized bleeding without significant hemoglobin reduction, hemodynamic instability, or life-threatening implications, TXA 1 g was administered orally four times daily and desmopressin IV 0.3 mg/kg every 12 h for three doses. In addition, alterations could be made based on the location of the bleeding. For example, basic pressure dressings, local topical TXA for mucosal bleeds, IV proton pump inhibitors for gastrointestinal bleeding, or nasal spray and packing for epistaxis. If the bleeding were to be more severe or systemic, IV TXA as well as IV Factor VIIa could be considered.

## Results

This regimen has been utilized for three different inpatients at LHSC since 2021 (Table 2); one patient had two transplants as part of the scheduled plan of care. All three were practicing Jehovah’s Witnesses who did not consent to most blood-derived products, including red blood cells and platelets. Two of the patients underwent salvage autologous HSCTs in the setting of relapsed/recurrent primary central nervous system diffuse large B cell lymphoma while the other patient received tandem HSCTs for high-risk multiple myeloma. None of the patients had a significant bleeding event, while all three patients did experience mild oral mucositis which was controlled with topical TXA. Furthermore, none had a hemoglobin nadir below 8.0 g/dL. All patients did have significant thrombocytopenias and neutropenias, including in both tandem HSCT procedures, but these resolved with TPO-RAs and G-CSF administration giving engraftment times comparable to “regular” autologous HSCTs. For any additional bleeding risk associated with thrombocytopenia, all patients received both oral and IV TXA. Two of the three patients also experienced febrile neutropenias and were treated with an appropriate course of antibiotics. All the cell lines of the patients had normalized by the time of discharge with all patients being hospitalized for <30 days, similar to the experience of our centre with “regular“ autologous HSCTs. All the patients are doing clinically well with the two lymphoma patients having an ongoing complete metabolic response.

**Table 2:** Jehovah’s witness patients who underwent London health sciences centre’s “bloodless” hematopoietic stem cell transplant (HSCT) protocol

Patient	1	2	3	
Age at treatment (years)	50	64	60	
Sex	Male	Female	Female	
Diagnosis	Relapsed primary CNS DLBCL with multifocal lesions	Recurrent primary CNS DLBCL	High risk MM	
Prior treatment	2019 MVPC with radiation and procarbazine maintenance 2020 Ibrutinib 2021 CHOP-R	2020 MPVC with radiation and procarbazine maintenance 2022 Ibrutinib	2022 CyBorD	
HSCT regimen (date, conditioning regimen, cell dose)	Autologous HSCT September 2021 Conditioning: Carmustine & Thiotepa Cell dose: 7.2 × 10^6^ CD34 cells/kg	Autologous HSCT November 2022 Conditioning: Carmustine & Thiotepa Cell dose: 3.8 × 10^6^ CD34 cells/kg	Autologous HSCT May 2023 Conditioning: Melphalan Cell dose: 6.2 × 10^6^ CD34 cells/kg	Autologous HSCT August 2023 Conditioning Melphalan Cell dose: 3.2 × 10^6^ CD34 cells/kg
Pretransplant Hb (g/dL)	15.7	13.3	9.0	12.7
Days to Hb Nadir	+13	+11	+13	+17
Hb Nadir (g/dL)	9.4	8.4	8.1	10.4
Days Hb <7.0 g/dL	0	0	0	0
Hb at discharge (g/dL)	12.0	10.4	9.9	13.2
Pretransplant Plt count (x 10^9^cells/L)	168	145	223	119
Days to Plt nadir	+9	+7	+10	+10
Plt nadir (x10^9^cells/L)	<5	<5	8	<5
Days Plt count <10 (x 10^9^ cells/L)	4	4	7	7
Plt count at discharge (x 10^9^ cells/L)	235	47	157	76
Pretransplant ANC (x 10^9^cells/L)	1.0	5.6	11.7	4.2
Days to ANC nadir	+2	+15	+6	+10
ANC nadir (x 10^9^cells/L)	0.0	2.5	0.0	0.2
Days ANC <0.5 × 10^9^cells/L	14	0	7	3
ANC at discharge (x 10^9^cells/L)	1.4	2.5	4.3	5.9
Days to neutrophil engraftment	+13	+11	+13	+13
Days to Plt engraftment	+13	+11	+21	+13
Filgrastim days administered	+5 to +12	+5 to +10	+5 to +16	+5 to +8
Days of grade 3–4 anemia	0	0	0	0
Days of grade 3 thrombocytopenia	7	5	0	4
Days of Grade 4 thrombocytopenia	1	4	7	11
Bleeding complications & interventions	No significant bleeding TXA given for low plt	No significant bleeding TXA given for low plt	No significant bleeding TXA given for low plt	No significant bleeding TXA given for low plt
Infection complications and interventions	Culture negative febrile neutropenia – imipenem until recovered ANC	None	Culture-negative febrile neutropenia – imipenem and vancomycin until recovered ANC	BCx neg febrile neutropenia – IV imipenem *C. difficile* colitis versus neutropenic colitis versus diverticulitis – PO vancomycin + metronidazole, bowel rest + parenteral nutrition Day +7 to +11
Other complications	Grade 2 mucositis with mild dysphagia and nausea – treated with koolstat, olanzapine (Zyprexa) and ondansetron Constipation – treated with desmopressin	Grade 2 mucositis with mild dysphagia – treated with koolstat, olanzapine (Zyprexa) and ondansetron	Grade 1 mucositis	None
Follow-up post-HSCT and clinical status	Complete metabolic response	Complete metabolic response	Pending follow-up	

Day 0: day of transplant; Hb: hemoglobin; Plt: platelet count; ANC: absolute neutrophil count; CNS: central nervous system; DLBCL: diffuse large B cell lymphoma; MM: multiple myeloma; MVPC: methotrexate (Methotrexate), vincristine, procarbazine, cyclophosphamide; CHOP-R: cyclophosphamide, hydroxydaunorubicin (doxorubicin), oncovin (vincristine), prednisone, rituximab; CyBorD: cyclophosphamide, bortezomib (Bortezomib), dexamethasone.

Epidemiologic information, diagnosis, prior treatments, HSCT regimen, cell count information, engraftment time, complications, and their interventions.

## Discussion

In summary, based on existing literature,^7^, ^8^, ^9^, ^10^, ^11^, ^12^, ^13^, ^14^, ^15^, ^16^ we developed a “bloodless” HSCT protocol (Table 1) with three Jehovah’s Witness patients undergoing treatment according to this protocol (Table 2). The protocol entailed pre-transplant targets for hemoglobin concentration >11.0 g/dL and platelet count >150 × 10^9^ cells/L achieved through ESAs, IV iron, and, when indicated, hormonal contraceptives. Our post-transplant regimen supported hematopoiesis with supplements including iron, vitamin B12, and folic acid as well as vitamin K for coagulopathy prevention. Hematopoiesis was additionally supported by injections of TPO-RAs and G-CSF. Unnecessary blood loss was prevented by decreasing the frequency and volume of blood draws to twice a week bloodwork using pediatric test tubes. Bleeding prevention was achieved via oral proton pump inhibitors, laxatives, and nasal spray. Response measures to severe anemias, thrombocytopenias, and bleeding were in place and entailed ESAs, TXA, iron, desmopressin, and tailored hemostasis measures. Of the three patients who underwent autologous HSCT with our “bloodless” protocol, none developed any significant bleeding event nor any severe anemia that would have necessitated blood product transfusion in regular HSCT protocols. While all the patients did develop severe thrombocytopenias, these were managed conservatively as outlined above and the patients did not develop any adverse outcomes (Table 2). These findings of safety of “bloodless” HSCTs are in line with previous experiences in other centres.^7^, ^8^, ^9^, ^10^, ^11^, ^12^, ^13^, ^14^, ^15^, ^16^ Therefore, both our review of the literature and own experiences demonstrate the feasibility of implementing “bloodless“ HSCTs with specialized and carefully planned protocols at Canadian centres.

The major limitation of this study is the small sample size of patients, an inherent issue given the niche population group being assessed; most of the previous literature represents small case series or retrospective analyses. However, in conjunction, there is a clear body of evidence showing that “bloodless” HSCTs can be done safely and with similar efficacy to “regular” HSCTs. This allows certain populations, especially Jehovah’s Witnesses, access to a previously unavailable treatment option. It is also important to note that negative findings in this study population are rarely published.

Moving forward, conducting further prospective trials would be beneficial to better elucidate the risks or disadvantages associated with “bloodless” protocols. Arranging such trials would be difficult given the small number of Jehovah’s Witnesses requiring HSCTs, especially in Canada, and would likely necessitate enrolling multiple centres to have a large enough population. As blood products are both a time-intensive and scarce resource, implementation of aspects of the “bloodless“ protocol for all patients may reduce the number of blood product transfusions required in HSCTs overall. A notable feature of this protocol is its applicability to other patients undergoing HSCT, with the objective of conserving blood products at our center. However, patients must be informed regarding the implications of declining transfusions, which include the possibility of clinical deterioration and the inability to provide support in critical situations such as life-threatening anemias and hemorrhages.

In conclusion, with careful planning and tailored regimens there is evidence supporting the achievability of “bloodless” HSCTs in patients, such as Jehovah’s Witnesses, who are not able to receive blood products through the course of their treatment. These advancements not only allow for practitioners to provide care to a previously excluded group but could minimize the use of resource scarce blood products in all HSCT patients.

## Author contributions

MM collected the data, framed the ideas, drafted, and critically revised the manuscript. AF and DC were responsible for data analysis, design of protocol, and manuscript review. UD designed the protocol, critically revised the manuscript, and supervised the project.

## Conflicts of interest

The authors declare no conflicts of interest.
